# Items

**Published:** 1903-10

**Authors:** 


### ITEMS.

The announcement is made of a work shortly to be published by Dr. G. Frank Lydston, entitled, The diseases of society, in which the questions of crime and the social evil will be discussed.

The Prize Essay of the American Medical Association.—The American Medical Association offers annually a gold medal, value of $100, for the best essay on any subject relating to medicine or surgery. The recipient of this prize will be given the option of the gold medal, or a bronze replica of the medal, and the balance of the appropriation (about $90) in money; or the entire amount ($100) in money. Inasmuch as the association annually sets apart the sum of $500 for original research, this prize is offered to stimulate the production of a superior practical paper based either upon experimental studies or clinical investigation, or both. The committee will give preference to papers having the greatest brevity consistent with thorough consideration of the subject, and recommends that the paper shall not exceed 5,000 words. The committee, while not restricting the choice of subjects, recommends as an important subject for consideration The therapeutic value of the digestive ferments. Competing essays must be typewritten, and bear no mark revealing their authorship; but instead of the name of the author there must appear on each essay a motto, and accompany each essay a sealed envelop containing the name of the author and bearing on its outer surface the motto of identification. No envelop will be opened by the committee until a decision has been reached as to the most deserving essay. The other essays will be returned to their respective authors. The committee reserves the right to reject all essays, if none are found worthy of the association medal. Competing essays must be in the hands of the chairman of the committee not later than April 1, 1904. Lewis S. McMurtry, Louisville, Ky., chairman; Burnside Foster, Saint Paul, Minn.; M. H. Fussell, Philadelphia, Pa., committee.

